# Effectiveness of Universal Self-regulation–Based Interventions in Children and Adolescents: A Systematic Review and Meta-analysis

**DOI:** 10.1001/jamapediatrics.2018.0232

**Published:** 2018-04-16

**Authors:** Anuja Pandey, Daniel Hale, Shikta Das, Anne-Lise Goddings, Sarah-Jayne Blakemore, Russell M. Viner

**Affiliations:** 1 University College London Great Ormond Street Institute of Child Health, London, United Kingdom; 2 Heriot-Watt University, Edinburgh, United Kingdom; 3 University College London Institute of Cognitive Neuroscience, London, United Kingdom

## Abstract

**Importance:**

Childhood and adolescence self-regulation (SR) is gaining importance as a target of intervention because of mounting evidence of its positive associations with health, social and educational outcomes.

**Objective:**

To conduct a systematic review and meta-analysis of rigorously evaluated interventions to improve self-regulation in children and adolescents.

**Data Sources:**

Keyword searches of the PsycINFO, PubMed, EMBASE, CINAHL Plus, ERIC, British Education Index, Child Development and Adolescent Studies, and CENTRAL were used to identify all studies published through July 2016.

**Study Selection:**

To be eligible for this review, studies had to report cluster randomized trials or randomized clinical trials, evaluate universal interventions designed to improve self-regulation in children and adolescents aged 0 to 19 years, include outcomes associated with self-regulation skills, and be published in a peer-reviewed journal with the full text available in English.

**Data Extraction and Synthesis:**

A total of 14 369 published records were screened, of which 147 were identified for full-text review and 49 studies reporting 50 interventions were included in the final review. Results were summarized by narrative review and meta-analysis.

**Main Outcomes and Measures:**

Self-regulation outcomes in children and adolescents.

**Results:**

This review identified 17 cluster randomized trials and 32 randomized clinical trials evaluating self-regulation interventions, which included a total of 23 098 participants ranging in age from 2 to 17 years (median age, 6.0 years). Consistent improvement in self-regulation was reported in 16 of 21 curriculum-based interventions (76%), 4 of the 8 mindfulness and yoga interventions (50%), 5 of 9 family-based programs (56%), 4 of 6 exercise-based programs (67%), and 4 of 6 social and personal skills interventions (67%), or a total of 33 of 50 interventions (66%). A meta-analysis evaluating associations of interventions with self-regulation task performance scores showed a positive effect of such interventions with pooled effect size of 0.42 (95% CI, 0.32-0.53). Only 24 studies reported data on distal outcomes (29 outcomes). Positive associations were reported in 11 of 13 studies (85%) on academic achievement, 4 of 5 studies on substance abuse (80%), and in all studies reporting on conduct disorders (n = 3), studies on social skills (n = 2), studies on depression (n = 2), studies on behavioral problems (n = 2), and study on school suspensions (n = 1). No effect was seen on 2 studies reporting on academic achievement, 1 study reporting on substance abuse, and 1 additional study reporting on psychological well-being.

**Conclusions and Relevance:**

A wide range of interventions were successful in improving self-regulation in children and adolescents. There was improvement in distal academic, health, and behavioral outcomes in most intervention groups compared with controls.

## Key Points

**Question:**

What is the effectiveness of universal self-regulation–based interventions to improve self-regulation and affect health and social outcomes in children and adolescents?

**Findings:**

This systematic review and meta-analysis of 49 randomized clinical trials evaluating 50 self-regulation interventions found that these interventions were effective in children and adolescents. Positive outcomes on health and social measures such as academic achievement, social skills, mental health, behavioral problems, conduct disorders, school suspensions, and substance abuse was also reported.

**Meaning:**

Self-regulation interventions can be effective in children and adolescents with possible benefits in health and social outcomes.

## Teaser

This systematic review and meta-analysis assesses the effectiveness and outcomes of interventions to improve self-regulation in children and adolescents.

## Introduction

Self-regulation (SR) is a psychological construct which encompasses a range of important competencies, including the capacity for controlling one's emotions, the ability to have positive interactions with others, the capacity for avoiding inappropriate or aggressive actions, and the ability to carry out self-directed learning.^1^ Cognitive processes contributing to SR are often referred to as executive functions, and they include the ability to direct or focus attention, shift perspective, and adapt flexibly to changes (cognitive flexibility); retain information (working memory); and inhibit automatic or impulsive responses to achieve a goal, such as problem-solving (impulse control).^2,3^ Self-regulation can be seen as the volitional administration of executive functions in the enactment of goal-related behavior.

There is growing evidence that SR plays an important foundational role in development and maintenance of physical health and well-being in childhood and across the lifespan.^4,5,6,7^ Greater SR has been associated with positive outcomes on a range of attributes, including school readiness, academic achievement, healthy behavior, physical health, and mental health. Conversely, poor SR has been linked to adverse outcomes, such as health risk behaviors, psychiatric disorders, substance dependence, crime, and unemployment.^8,9,10,11,12,13^

Growing evidence of the importance of SR to improve life chances has driven the development of a number of interventions designed to improve SR skills during childhood and adolescence. An increasing number of interventions have shown promise in randomized clinical trials of self-regulatory capacity improvements, ranging from playgroup games to training in yoga and martial arts. Yet it remains unclear which type of interventions are most effective and if effectiveness differs by population, setting, or intervention characteristics. Previous attempts to synthesize evidence on self-regulation interventions have been limited to target populations (eg, people with attention-deficit/hyperactivity disorders) and specific age-groups (eg, children age 0-10 years).^14,15^ No review to date has evaluated the effectiveness of self-regulation interventions in children and adolescents (age 0-19 years) and examined the effects of such interventions on distal health and social outcomes.

We undertook a systematic review of the effectiveness of universal interventions designed to promote SR in children and adolescents (age 0-19 years), summarizing both evidence on effectiveness of such interventions in improving SR and on improving distal health and social outcomes, such as academic achievement, substance abuse, well-being, and so on.

## Methods

**Figure 1.**
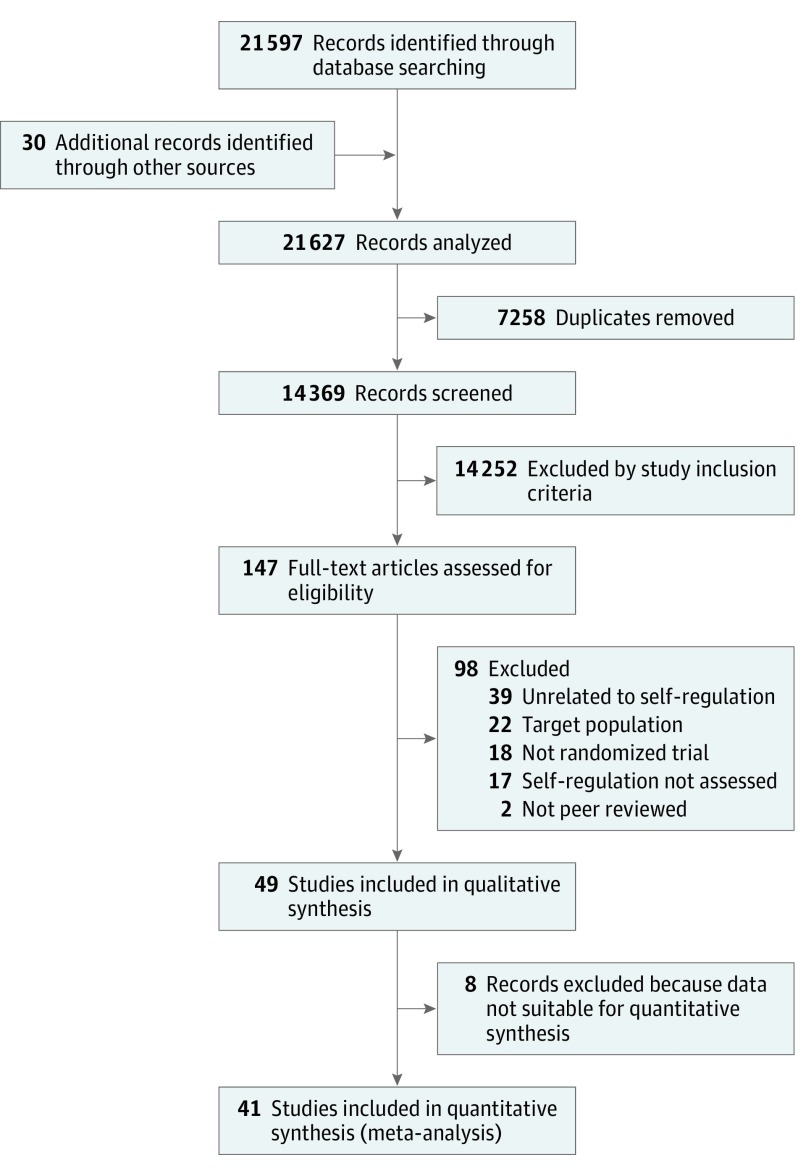
Preferred Reporting Items for Systematic Reviews and Meta-analysis Flow Diagram

We followed an a priori search protocol to identify randomized and cluster randomized clinical trials that evaluated interventions focused on SR in children and adolescents. The primary search included 8 electronic databases: MEDLINE (PubMed), PsycINFO, Excerpta Medica database (EMBASE) via Ovid; Educational Resources Information Center (ERIC), Cumulative Index to Nursing and Allied Health Literature (CINAHL) Plus, British Education Index, Child Development and Adolescent Studies via EBSCO, and Cochrane Controlled Trials Register (CENTRAL) in July 2016 to identify potential studies for inclusion. In addition, we also searched reference lists and citations of included studies to identify any additional studies. The details of the electronic search results are summarized in Figure 1.

One reviewer (A.P.) ran the preliminary search and screened abstracts of 14 369 eligible studies for inclusion. A 10% proportion of the total titles and abstracts were randomly selected and screened independently by a second reviewer (D.H.). Results were compared to ensure that there was less than 5% discrepancy in the results, after which screening of all search results was completed by 1 reviewer (A.P.). After screening, 147 articles were identified for full-text review. These articles were reviewed independently by 2 reviewers (A.P. and D.H.) for inclusion. Any discrepancy was resolved by consulting a third reviewer (R.M.V.).

Studies were eligible for the review if they (1) reported randomized clinical trials and cluster randomized trials; (2) evaluated universal interventions designed to improve SR in children and adolescents (age 0-19 years); (3) included at least 1 child-based outcome associated with SR skills; and (4) was a peer-reviewed publication published in the English language. All studies from the beginning date of database archives were eligible for inclusion.

We used a standardized data extraction form to record information about each study, including general information consisting of authors, country, study design, randomization procedure; population characteristics consisting of age, sex distribution, ethnicity, any special demographics, baseline imbalances, withdrawals, and exclusions; and intervention characteristics consisting of content, mode of delivery, timing, intervention provider, and fidelity of intervention. The interventions could be classified into 5 categories: curriculum interventions, physical activity and exercise interventions, mindfulness and yoga interventions, parenting and family-focused interventions, and other skills-based training.

We gathered information in detail about SR skills, which was our primary outcome of interest, including tools used, assessors, presence of blinding in assessment, and validity of the assessment tools. A number of different outcome sources were included overall, such as parent, teacher, self- reports, and task-based measures. Findings for SR outcome were quantified by effect sizes and CIs for quantitative synthesis. We also gathered information on distal health and social outcomes, including academic achievement, psychological stress, and substance abuse.

Quality assessment was conducted using the Effective Public Health Practice Project Quality Assessment Tool for Quantitative Studies.^16^ Included studies were assessed with regards to selection bias, study design, confounders, blinding, data collection methods, withdrawals, dropouts, intervention integrity, and analyses.

### Statistical Analysis

Of 50 included interventions, relevant data for quantitative synthesis were available for 42. Effect sizes were calculated for the intervention group relative to the comparison group for each study. We used the Cohen *d* (defined as the difference between posttest means divided by the pooled standard deviation) as the effect size index. When 2 studies reported analyses of the same sample, we included the larger sample for meta-analysis. Wherever available, descriptive statistics (mean, standard deviation, and sample size) was used to estimate effect size. Wherever such information was unavailable, we estimated effect sizes from available inferential statistics. When more than 1 outcome variable associated with SR was reported, we attempted to use the summary statistic for all aspects of SR. When such summary statistics were unavailable, we selected the outcome variable that best described SR. All effect sizes were calculated such that positive scores indicate a desirable outcome for intervention participants compared with control participants. All but 1 study reported continuous outcomes.^17^ We conducted all analysis using an effect size calculator.^18^ Meta-analysis was performed using a random-effects model.^19^ We assessed for publication bias by visual inspection of funnel plots. The *I*^2^ statistic was calculated as a measure of heterogeneity, which is interpreted as the proportion of total variation in the estimated treatment effect that arises from between-study heterogeneity rather than by chance.^20^ We also conducted subgroup analyses to explore for sources of heterogeneity based on the age of study participants, the type of intervention, the duration of the intervention, and the source of outcome measure.

This review is registered in PROSPERO (registration number CRD42016047661) and further details on methodology can be found in the published protocol.^21^ We report our results in the form of a narrative review for all included studies and a meta-analysis for studies including data that permitted a quantitative synthesis. Findings are reported in accordance with the Preferred Reporting Items for Systematic Reviews and Meta-analysis statement.^22,23^

## Results

Our literature search strategy identified 14 369 potentially relevant studies; 49 studies reporting 50 interventions met all eligibility criteria and were included in the final review (including a total of 23 098 participants, a mean of 462 participants per study). Our review included records published over a span of 4 decades (1977-2017). On assessment, 3 studies were rated as high quality, 38 as moderate in quality, and 8 as poor quality. The most common reason for downgrading a quality rating was a lack of blinding of participants, which was difficult to achieve in this type of intervention; 46 studies (93.8%) were downgraded for this reason. Detailed characteristics of included studies^5,17,24,25,26,27,28,29,30,31,32,33,34,35,36,37,38,39,40,41,42,43,44,45,46,47,48,49,50,51,52,53,54,55,56,57,58,59,60,61,62,63,64,65,66,67,68,69,70,71,72,73,74^ are summarized in eTables 1-5 in the Supplement.

The age of study participants ranged from 2 to 17 years, with a median of 6.0 years. Although we identified interventions evaluated for children in the age group 0 to 2 years in our literature search, they were limited to target populations, and reports on universal interventions were only available for children older than 2 years. The pooled sample characteristics including sex, location, study setting, socioeconomic status, and race/ethnicity are described in the Table.

**Table.** Pooled Sample Descriptive Characteristics

Participant Features	No. (%) of Participants
Educational level of participants	
Preschool	6566 (28.4)
Primary/ elementary school (grades 1-5)	13 403 (58.0)
Middle school (grades 6-8)	2928 (12.7)
High school (grades 9-12)	201 (0.87)
Sex	
Male	11 784 (51.0)
Female	9066 (39.3)
Not reported	2248 (9.7)
Geographical area/study setting	
Urban	2655 (11.5)
Suburban	99 (0.43)
Rural	2295 (9.9)
Not reported	10 031 (43.4)
Multiple	8018 (34.7)
Country	
United States	19 583 (84.8)
Canada	170 (0.7)
Australia	65 (0.3)
Switzerland	181 (0.8)
United Kingdom	98 (0.4)
Italy	75 (0.3)
Belgium	47 (0.2)
Spain	186 (0.8)
China	87 (0.4)
Chile	1876 (8.1)
Ireland	730 (3.2)
Participant socioeconomic status	
Low income^a^	7929 (34.3)
Middle/high income	9677 (41.9)
Not reported	5492 (23.8)
Race/ethnicity	
Hispanic	2017 (8.7)
White	8297 (35.9)
African American	6681 (28.9)
Native American	33 (0.1)
Asian/Asian American	687 (3.0)
Mixed/other	1521 (6.6)
Not reported	3862 (16.7)

^a^ Low-income children and adolescents were defined as those described in published studies as receiving free school lunches, receiving federal benefits, or living in families whose income was less than the federal poverty level.

The studies used validated tools for assessment of SR, such as the Behavior Rating Inventory of Executive Function,^57^ Humphrey Self-control inventory,^56^ Devereux Early Childhood Assessment,^24^ and Social Control Questions of the Social Skills Rating System^48,59^ for parent reports, teacher reports, and self-reports of self-regulated behavior. For directly assessed SR skills quantified by scoring participants on task performance, the Head, Toes, Knees, and Shoulders task and ^25,29^ Flanker and reverse Flanker tasks^26,30,41,44,47,58,73^ were most commonly used.

### Narrative Review

The SR interventions were classified as curriculum based, mindfulness and yoga, family based, exercise based, and other social and personal skills–based intervention strategies/delivery methods. These classifications were based on discussion between the review team members (A.P., D.H., and S.D.). The interventions were delivered by teachers, psychologists, yoga trainers, parent consultants, research facilitators, and/or staff trained in a specific curriculum or specialty area.

### Curriculum-Based Interventions

**Figure 2.**
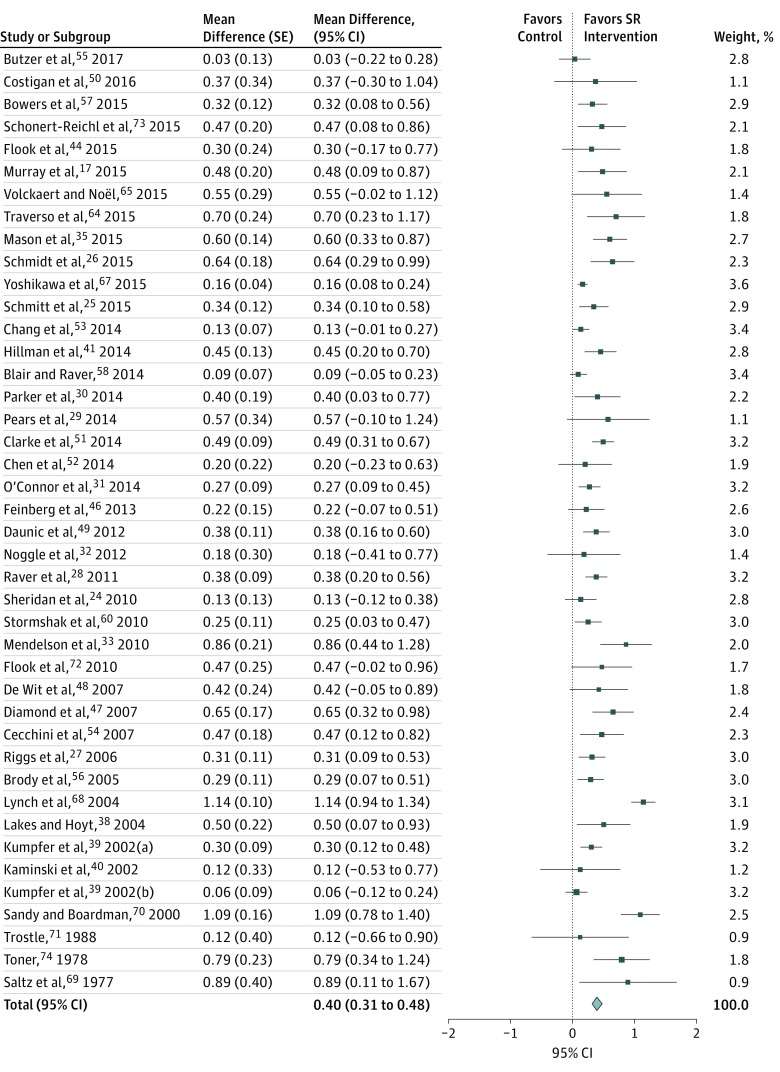
Forest Plot of Intervention Results Data were pooled under the assumption of a random-effects model; τ^2^ = 0.05; χ^2^_41_ = 178.14; *P* < .001; *I*^2^ = 77%; overall effect z = 9.16; *P* < .001. Kumpfer et al^39^ includes 2 separate analyses. SR indicates self-regulation.

Curriculum-based interventions were the most common type of intervention used to enhance SR, especially for the age groups younger than 10 years. Twenty-one of 50 interventions (42%) used a curriculum-based approach implemented in classrooms, usually with teachers as one of the main intervention providers. These interventions included a combination of teachers’ professional training and classroom-based activities based on a predefined curriculum. They were mainly based in preschool (n = 12 of 21 [57%]),^5,25,28,29,40,47,58,59,63,67,68,70^ primary school (n = 7 of 21 [33%]),^27,31,39,42,49,51,66^ middle school (n = 1 of 21 [5%]),^36^ and high school (n = 1 of 21 [5%])^57^ settings, and the intervention was embedded within the existing school routine. Some of these (n = 8 of 21 [38%])^28,39,42,49,51,58,66,67^ interventions were evaluated on large samples (more than 500 participants). The strategies used in the preschool and kindergarten age group included circle-time games, storytelling, book reading, and self-talk; in older children, the curriculum included activities such as role play, cognitive modeling, and psychoeducational group therapeutic lessons. They typically had implementation support (eg, coaching or supervision) to promote fidelity (ie, the extent to which the intervention was delivered as intended), but fidelity was infrequently measured and reported (n = 11 of 21; 52%).^5,24,25,30,46,47,49,51,63,66,73^ Of 21 interventions, 16 (76%)^5,25,27,28,31,36,39,47,49,51,57,59,66,67,68,70^ reported consistent improvements in SR in the intervention group compared with the control group. Of 10 studies (48%)^27,28,29,31,36,57,58,59,63,67^ reporting on academic achievement, 8 (38%)^27,28,29,31,36,57,58,63^ showed consistent improvements. There was also improvement reported in social skills,^51^ conduct,^66^ and behavioral problems^31^ in intervention participants compared with control participants (1 study each [5%]). eTable 1 in the Supplement gives details on these studies, and Figure 2 shows the forest plot of effect sizes of these interventions.

### Physical Activity/Exercise-Based Interventions

A total of 6 studies^26,38,41,50,52,54^reported interventions using physical activity or exercise to promote SR. These interventions were mainly evaluated on the preadolescent (n = 2 of 6 [33%])^41,52^ and adolescent (n = 3 of 6 [50%])^26,50,54^ age groups; the sixth was an intervention of children in kindergarten through grade 5.^38^ These interventions used techniques such as high-intensity interval training (n = 1 of 6 [17%]),^50^ martial arts (n = 1 of 6 [17%]),^38^ and team games (n = 4 of 6 [67%]).^26,41,52,54^ They were delivered in school or after-school recreational facility settings and involved specialized staff (trained professionals) for implementation. Four^26,38,41,54^of 6^26,38,41,50,52,54^ interventions (67%) reported higher SR scores in the intervention groups compared with the control groups, which was significant on conventional tests of statistical significance. One study^38^ (17%) reporting on a martial arts intervention showed statistically significant improvement in academic achievement in the intervention group compared with controls. While 1 study^50^ reported the association of interventions with psychological well-being, this study did not meet the threshold for statistical significance.

### Mindfulness/Yoga Interventions

Eight studies^30,32,33,44,45,55,72,73^ evaluated mindfulness and/or yoga techniques to enhance SR. These interventions were mostly applied within the adolescent age group, and although the effect on long-term outcomes was not studied in most interventions, these interventions did show promise in improving SR in adolescents. All the interventions were of short duration (6 months or less) and school based, with qualified mindfulness or yoga instructors and assistants delivering the intervention. Mindfulness and yoga and exercise-based interventions were evaluated and found to be effective especially for the preadolescent and adolescent age groups. Four^30,33,45,73^of the 8 interventions (50%) produced a statistically significant effect size favoring the intervention group. The sample size of evaluated interventions was small, and that might be one of the reasons why the proportion of interventions producing a statistically significant difference was relatively low compared with other types of interventions. One intervention each (13%) showed benefits in academic achievement,^44^ substance use,^55^ and mental health^73^ in active-arm participants compared with control participants.

### Family-Based Intervention

Our review identified 9 interventions^24,34,39,43,46,48,53,56,60^ in which parents and/or siblings were an important part of the intervention to enhance SR. These interventions were useful for different age groups across childhood and adolescence. The strategies evaluated included group meetings (n = 1 of 9 [11%]),^24^ skill building of parents and children (n = 3 of 9 [33%]),^34,39,56^ after-school programs with siblings (n = 1 of 9 [11%]),^46^ mentoring (n = 1 of 9 [11%]),^48^ and parent consultation (n = 3 of 9 [33%]).^43,53,60^ More than half of them were community based (n = 5 of 9 [56%]),^24,34,48,53,56^ while others were school based (n = 4 of 9 [44%]).^39,43,46,60^ Compared with other interventions, these interventions had longer follow-up durations. Family-based interventions were successful in bringing a consistent change in SR measures in intervention groups in 5 of the 9 studies (56%).^34,39,43,56,60^ There was also benefit reported in distal outcomes, with better academic achievement,^46^ mental health,^60^ social skills,^34^ and behavioral problems^43^ in 1 study each (11% each); conduct problems in 2 studies (22%)^34,56^; and reduction in substance use in 3 studies (33%).^34,43,56^

### Other Social and Personal Skills

**Figure 3.**
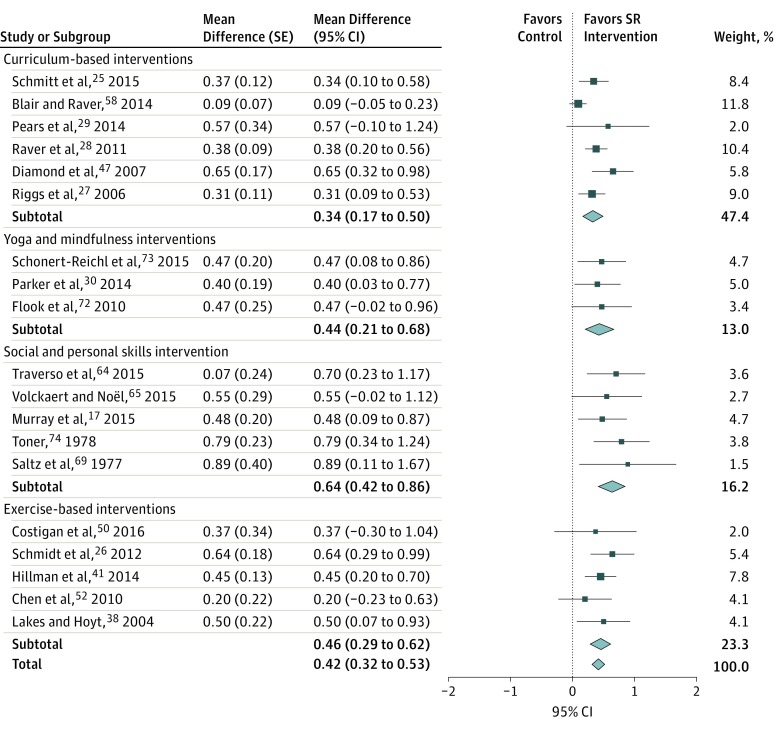
Forest Plot of the Effect Sizes of Self-regulation Task Performance Scores Data were pooled under the assumption of a random-effects model. For curriculum-based interventions, τ^2^ = 0.02; χ^2^_5_ = 14.35; *P* = .01; *I*^2^ = 65%; overall effect z = 4.04; *P* < .001. For yoga and mindfulness interventions, τ^2^ = 0.00; χ^2^_2_ = 0.08; *P* = .96; *I*^2^ = 0%; overall effect z = 3.66; *P* < .001. For social and personal skills interventions, τ^2^ = 0.00; χ^2^_4_ = 1.61; *P* = .81; *I*^2^ = 0%; overall effect z = 5.74; *P* < .001. For exercise-based interventions, τ^2^ = 0.00; χ^2^_4_ = 2.50; *P* = .64; *I*^2^ = 0%; overall effect z = 5.41; *P* < .001. In total, for curriculum-based interventions, τ^2^ = 0.02; χ^2^_18_ = 30.85; *P* = .03; *I*^2^ = 42%; overall effect z = 8.16; *P* < .001. For subgroup differences, χ^2^_3_ = 4.94; *P* = .18; *I*^2^ = 39.3%. SR indicates self-regulation.

A final subgroup of 6^17,64,65,69,71,75^ evaluated interventions included social and personal skills training in small groups using frameworks of personal responsibility, model behavior, conflict resolution, and so on. These were especially useful in aspects of SR such as delay of gratification, effortful control, and attention. These interventions were highly effective, with only 2^65,71^ of the 6 studies (33%) reporting a small and statistically insignificant change, while others reported^17,64,69,75^ significant improvements in SR measures (Figure 3). These interventions were focused on the aspects of SR and mostly tested the skills on task-based performance scores. The relatively high effect size of these interventions (*d*, 0.64; 95% CI, 0.42-0.86 vs an overall *d* of 0.42; 95% CI, 0.32-0.53) can be explained by the focused nature of these interventions on aspects of SR, and testing skills on task-based performance scores. None of these studies reported on health and social outcomes.

### Meta-analysis

We first examined the overall effect on SR outcomes based on effect size data from the evaluation of 42 interventions that qualified for meta-analysis. These interventions included 13 405 participants. There was heterogeneity in the outcomes, as indicated by poor overlap of effect size CIs and a high *I*^2^ statistic (77%); hence drawing interpretations from the quantitative synthesis of all studies combined was impossible (Figure 2). We explored for sources of heterogeneity by conducting a meta-regression with age, socioeconomic status, type of intervention, and outcome assessment outcomes (including self-reported, teacher-reported, parent-reported, and task performance–based outcomes). However, we could not identify any significant associations. We conducted subgroup analysis and found that the heterogeneity was partly accounted for by the assessment methods used to measure SR (ie, whether SR was measured by parent report, teacher report, or self-report of SR behavior or was assessed based on participants’ performance scores on tasks demonstrating SR skills). We found that restriction of analysis to the studies that measured SR through performance testing requiring SR skills reduced the *I*^2^ score from 77% to 39.3% (Figure 3).

In addition, the parent-reported, teacher-reported, and self-reported scores are subjective assessments, while task-based scores are direct measurements of SR skills and hence less likely to be affected by detection bias. Thus, in our final meta-analysis, we only included studies reporting SR outcomes based on task performance scores. We tested these studies for publication bias by creating a funnel plot (eFigure in the Supplement). The funnel plot was symmetrical, indicating that publication bias in included studies was unlikely. Next, we assessed the effectiveness of interventions from these 19 studies by pooling the results on a forest plot (Figure 3). We found that the overall effect was statistically significant and favored the intervention (pooled effect size *d* = 0.42; 95% CI, 0.32-0.53). Social and personal skills intervention had a higher mean effect size (*d* = 0.64; 95% CI, 0.42-0.86). There was considerable heterogeneity in data outcomes from studies using parent-reported, teacher-reported, and self-reported measures of SR(*I*^2^ = 77%); as a result, a meta-analysis for the outcomes in these studies was not undertaken.

### Effects on Health and Social Outcomes

Data regarding the effect of interventions on concurrent and distal health and social outcomes were found in 24 of 49 studies (49%). The effect of SR interventions on these outcomes is summarized in eTable 6 in the Supplement. Quantitative synthesis of effect sizes for these outcomes was precluded by the small number of studies reporting the data required to calculate effect sizes, and considerable heterogeneity in outcome measures. The follow-up period for these interventions ranged from 3 months to 5 years.

The outcome studied most commonly was academic achievement, and 11^27,28,29,31,36,38,44,46,57,58,63^ of 13 studies^59,67^ reported a positive impact in the SR intervention group, which was statistically significant. The areas of academic achievement with improvement included literacy, mathematics, reading, letter naming, and vocabulary. One study reported no impact,^67^ and another reported a marginal improvement that was not significant on statistical analysis.^59^ Three studies studied the effect on conduct disorders and found fewer conduct problems in the intervention groups compared with the control groups (eTable 6 in the Supplement).^35,56,66^ In 1 of the studies^61^ in which further analysis was done, it was observed that the prevention effects were stronger for youth at greater risk of developing conduct problems. Social skills, behavioral problems, and mental illness (depression) were studied in 2 studies each, and a positive impact was observed with respect to all outcomes.^31,43,48,51,60,73^ One study reported a decrease in number of school suspensions after intervention,^35^ and 4^35,43,55,56^ of 5 studies on substance use observed a benefit in the intervention group; the fifth study^30^ showed no benefit. A small but statistically insignificant improvement was seen on 1 study^50^ reporting on psychological well-being. There was no advantage of 1 type of intervention over another with regards to effect on distal outcomes.

## Discussion

Findings from the current systematic review demonstrate overall effectiveness of SR interventions in children and adolescents. A number of rigorously evaluated interventions for SR using different types of interventions were found to be effective. By summarizing the evidence on studies evaluating SR interventions, we observed that while most interventions (n = 33 [66%]) were successful in improving SR, some of them did not produce a noticeable change (n = 17 [34%]). When synthesizing the evidence quantitatively, we found considerable heterogeneity in the outcomes, and thus it may be difficult to draw conclusions on effectiveness based on pooled effect size estimates. However, based on the number of effective interventions, it can be concluded that each intervention type was effective in most of the studies testing them. The interventions were effective across all child and adolescent age groups and in both community and school settings. Curriculum-based approaches were most commonly used, and these involved training components for teachers who then implemented these interventions in classrooms. Compared with curriculum interventions, mindfulness and yoga interventions were shorter in duration but required additional staff in the form of trained yoga and exercise instructors. Family-based interventions used factors such as parenting practices and sibling relationships to enhance SR. Despite some of these interventions being based in communities, where it is a challenge to recruit and retain participants, the studies were able to achieve good compliance and effectiveness. The social and personal skills interventions were successful in improving SR through model behavior, attention training, and fantasy play activities in small groups. The pooled effect size for interventions reporting SR improvement on task performance scores was small, but comparable with other reviews looking at universal intervention for children and adolescents.^75^

There was evidence mainly on the effect on distal outcomes (academic achievement, mental health, social skills, and frequency of school suspensions), better educational attainment, and lower tendency for substance abuse in intervention groups compared with controls. One study found that the intervention effect was highest for those at greater risk for conduct problems,^61,62^ thus offering the promise of interventions where they are needed most.

There was no particular age group in which interventions were most effective; they improved SR scores across all age groups. Although most of the studies were conducted in the United States, there was also racial/ethnic diversity in the study subjects because some studies were conducted in areas with racial/ethnic minority populations or mixed populations. Our review included 7929 low-income study participants (34.3%), which showed efficacy of interventions in a disadvantaged group, including that outcomes such as academic success and employment are harder to achieve. We also observed that SR can be improved in childhood as well as in adolescence, thus providing an extended window of opportunity to intervene and improve outcomes.

The findings of this review would be useful for policy makers, educators, and health professionals focusing on prevention as SR attracts more attention as an intervention target. The findings can be a useful aid when designing SR interventions, with a range of effective intervention strategy options available. Also, some interventions brought more improvements in SR measures of those with lower baseline scores.^43,63^ Such interventions can be particularly useful when limited resources are available, and there is need to design interventions for the most vulnerable children and adolescents. Curriculum-based interventions were delivered in schools by training of teachers without a need for considerable additional resource in terms of time and staff. In addition, children and adolescents spend a considerable part of their time at school between the ages of 5 and 18 years and thus are easily accessible for interventions. Considering these factors, curriculum-based interventions can be preferred over other types of interventions. The effect of SR interventions on outcomes such as educational attainment, substance abuse, conduct problems, mental health, school suspensions, and behavioral problems shows promise for SR as an intervention target that can help improve educational, health, and social outcomes and thus provide opportunities to reduce inequalities in these areas.

Our study has a number of strengths, including being, to our knowledge, the first comprehensive review and meta-analysis of rigorously evaluated SR interventions in children and adolescents. Also, most of the included studies were moderate to strong quality in evidence.

### Limitations

There are limitations to this systematic review that need consideration. The SR outcome measures were not uniform, and there was substantial heterogeneity in their reporting. Considering this limitation of the evidence base of SR interventions, we would recommend that future research in SR should be directed to evaluate standard methods of reporting SR outcomes. Our review was also limited to published literature in the English language. We may have therefore missed unpublished work and studies published in other languages. The generalizability of our research findings may also be affected by the substantial number of studies conducted in the United States.

## Conclusions

In conclusion, our study findings suggest that SR interventions are effective and that improvements in educational, health, and social outcomes can follow improvements in SR. Different types of interventions can be used to improve self-regulation, and many of these strategies appear effective.

## Notes

Supplement. **eTable 1.** Characteristics of Curriculum based interventions including population description, setting and intervention details. **eTable 2.** Characteristics of Family based interventions including population description, setting and intervention details. **eTable 3.** Characteristics of mindfulness & yoga interventions including population description, setting and intervention details. **eTable 4.** Characteristics of exercise based interventions including population description, setting and intervention details. **eTable 5.** Characteristics of social and personal skills interventions including population description, setting and intervention details. **eTable 6.** Effect of self-regulation interventions on health and social outcomes : Name of Intervention, Outcomes and results. **eFigure.** Funnel Plot for pooled analysis of effect sizes of self-regulation task performance scores.
