# Supplementary material for: Effectiveness of Universal Self-regulation–Based Interventions in Children and Adolescents: A Systematic Review and Meta-analysis
Source: JAMA Pediatr. 2018 Apr 16;172(6):566–75. doi: 10.1001/jamapediatrics.2018.0232 (PMC6059379; doi:10.1001/jamapediatrics.2018.0232)
Supplement: Supplement. — eTable 1. Characteristics of Curriculum based interventions including population description, setting and intervention details. eTable 2. Characteristics of Family based interventions including population description, setting and intervention details. eTable 3. Characteristics of mindfulness & yoga interventions including population description, setting and intervention details. eTable 4. Characteristics of exercise based interventions including population description, setting and intervention details. eTable 5. Characteristics of social and personal skills interventions including population description, setting and intervention details. eTable 6. Effect of self-regulation interventions on health and social outcomes : Name of Intervention, Outcomes and results. eFigure. Funnel Plot for pooled analysis of effect sizes of self-regulation task performance scores. [file jamapediatr-172-566-s001.pdf]

Supplementary Online Content

Pandey A, Hale D, Das S, Goddings A-L, Blakemore S-J, Viner R. Effectiveness of universal self-regulation–based interventions in children and adolescents: a systematic review and meta-analysis. *JAMA Pediatr.* Published online April 16, 2018. doi:10.1001/jamapediatrics.2018.0232.

eTable 1. Characteristics of Curriculum based interventions including population description, setting and intervention details

eTable 2. Characteristics of Family based interventions including population description, setting and intervention details

eTable 3. Characteristics of mindfulness & yoga interventions including population description, setting and intervention details

eTable 4. Characteristics of exercise based interventions including population description, setting and intervention details

eTable 5. Characteristics of social and personal skills interventions including population description, setting and intervention details

eTable 6. Effect of self-regulation interventions on health and social outcomes : Name of Intervention, Outcomes and results

eFigure. Funnel Plot for pooled analysis of effect sizes of self-regulation task performance scores.

This supplementary material has been provided by the authors to give readers additional information about their work.

© 2018 Anuja Pandey, MD, et al. *JAMA Pediatrics.*

eTable 1: Characteristics of Curriculum based interventions including population description, setting and intervention details

Sr. no.	Study ID	Country	Intervention	Population Characteristics	Intervention description	Intervention aim/theory	Duration	Setting	Providers
1	Barnett 2008	United States	Tools of the Mind	274 (Tools=106, Control= 168) 3 & 4 yr. old children in low income urban school dist.	40 Vygotsky-inspired activities designed to promote mature dramatic play, self-regulatory private speech & facilitate attention and memory	SR, teaching mathematics and literacy skills with focus on play	1 yr.	Classroom	Trained Teachers
2	Diamond 2007	United States	Tools of the Mind	147 pre-schoolers (Tools=85, Control=62) in low-income, urban school dist.	40 Vygotsky-inspired activities designed to promote mature dramatic play, self-regulatory private speech & facilitate attention and memory	SR, teaching mathematics and literacy skills with focus on play	1 yr.	Preschool	Trained Teachers
3	Blair 2014	United States	Tools of the Mind	759 children in 79 kindergarten classrooms	40 Vygotsky-inspired activities designed to promote mature dramatic play, self-regulatory private speech & facilitate attention and memory	SR, teaching mathematics and literacy skills with focus on play	1 yr.	Kindergarten	Trained Teachers
4	Bowers 2015	United States	Student Success Skills (SSS) Program	201 (SSS=107, Control=94) 8th grade students(12-15 yrs.) at a rural school	SSS psychoeducational group classroom guidance lessons, 1 lesson per wk., for 5 wks.	self-regulatory development, school connectedness	5 wks.	Classroom	Trained School Counsellors

© 2018 Anuja Pandey, MD, et al. *JAMA Pediatrics.*

5	Lemberger 2015	United States	Student Success Skills (SSS) Program	193 middle school students, predominantly Hispanic, economically challenged school dist.	SSS classroom guidance lessons, 1-hr. lesson per wk., for 5 wks.	executive function, school connectedness	5 wks.	School	Trained School Counsellors
6	Bierman 2008	United States	HeadStart REDI	356 children in 44 Head Start classrooms, ethnically diverse, Age: Mean=4.49 yrs., SD=0.31	Pre-school PATHS Curriculum, centre based extension activities, training to support generalized skill development and home based learning.	SR, language/emergent literacy skill development, school readiness	1 yr.	School	Trained Teachers
**Sr. no.**	**Study ID**	**Country**	**Intervention**	**Population Characteristics**	**Intervention description**	**Intervention aim/theory**	**Duration**	**Setting**	**Providers**
7	Clarke 2014	Ireland	Zippy's Friends	730 children; Mean age 7 yrs., 3 months, 67% rural.	24 sessions divided into six modules, each with six illustrated stories involving an imaginary stick insect called Zippy	SR, emotional wellbeing	1 yr.	School	Trained teachers, Health Promotion Specialists
8	CPPRG 1999b	United States	Fast Track version of PATHS (Universal component)	6715 Grade 1 children , 55% receiving free school lunch,49% ethnic minority (African American)	57-lesson social competence intervention focused on self-control, emotional awareness, peer relations, and problem solving	self-control	3 yrs.	School	Trained teachers & educational coordinators
9	Riggs 2006	United States	PATHS curriculum	318 2nd and 3rd grade students aged 7-9 yrs. in four schools in the Seattle	20-30 min lessons , 3 times/wk. with focus to provide youth with opportunities to practice conscious strategies for self-control	inhibitory control, social competence	1 yr.	School	Trained teachers

© 2018 Anuja Pandey, MD, et al. *JAMA Pediatrics.*

10	Daunic 2012	United States	Tools for getting along (TFGA)	1296 students from 4th & 5th grade in 14 schools, 53.9% participants White; 34.8% participants African American	27 lesson intervention including cognitive modelling, role-plays, small group activities, and explicit application of strategies to real life social scenarios.	executive function, social problem solving	1 yr.	School	Trained teachers and guidance counsellors
11	Kumpfer 2002	United States	I Can Problem Solve (ICPS)	1st graders from 12 rural schools, primarily Caucasian (87%) and Hispanic (7.6%).	28 sessions of Games, stories, puppets, and role playing of 20 min. each integrated in classroom curriculum	SR, problem solving	1 yr.	School	Trained teachers
12	Kaminski 2002	United States	Project STAR	146 4 yr. old children	Teacher Training, The Parent Education and Support, & Home Visiting components	SR, parenting skills, family management	2 yrs.	School	Trained teachers, project staff
**Sr. no.**	**Study ID**	**Country**	**Intervention**	**Population Characteristics**	**Intervention description**	**Intervention aim/theory**	**Duration**	**Setting**	**Providers**
13	Lynch 2004	United States	Al's Pals: Kids Making Healthy Choices Curriculum	17 intervention classrooms (n =218) & 16 control classrooms (n = 181), mean age =52 mos.	2 lessons of 15-20 min each per wk., designed to promote social and emotional competence in young children.	self-control	23 wk.	School	Trained teachers
14	O Connor 2014	United States	INSIGHTS Into Children's Temperament (INSIGHTS)	435 kindergarten students from schools serving low income neighbourhoods	Classroom progams with teacher & parent components (2 hr. weekly sessions)to support children's SR by enhancing their attentional and behavioural repertoire	SR, attention, behaviour problems, academic achievement	10 wks.	School	Trained facilitators with graduate degree in psychology

© 2018 Anuja Pandey, MD, et al. *JAMA Pediatrics.*

15	Pears 2014	United States	Kids in Transition to School (KITS)	39 children approx. 5 yrs. of age	The KITS intervention includes weekly homework assignments, weekly Home–School Connection newsletters outlining the school readiness group topics for a given week, and home practice activities	SR , school readiness	24 wks.	School	Trained group teachers and parent group facilitators
16	Raver 2011	United States	Chicago School Readiness Project (CSRP)	602 children aged 3–4 from 35 classrooms, low income	Teachers training, ongoing classroom-based and child-focused consultation & stress reduction workshops for teachers	SR	1 yr.	Preschool	Trained teacher & mental health consultant
17	Sandy Boardman 2000	United States	Peaceful Kids Early Childhood Social-Emotional Learning (ECSEL) Program	404 children , ages 2.5 to 6 yrs.	15 once-a-wk. circle time session for children & 4 workshops for parents to promote social-emotional, cognitive, and conflict resolution skills development	self-control, conflict resolution	5 mos.	Day care & Head Start centre	Teacher, Teaching Assistant & aide
**Sr. no.**	**Study ID**	**Country**	**Intervention**	**Population Characteristics**	**Intervention description**	**Intervention aim/theory**	**Duration**	**Setting**	**Providers**

© 2018 Anuja Pandey, MD, et al. *JAMA Pediatrics.*

18	Tominey 2011	United States	Red Light, Purple Light	65 preschool children	16 sessions of 30 min each with playgroup games like Red light, purple light, freeze game, colour matching freeze, sleeping, conducting orchestra, drum beats held twice weekly	SR	8 wks.	Preschool	2 assistant teachers, researcher
19	Schmitt 2015	United States	Red Light, Purple Light	276 children/ 14 classrooms; Mean age = 51.69 mos., SD = 6.55	16 sessions of 30 min each with playgroup games like Red light, purple light, freeze game, colour matching freeze, sleeping, conducting orchestra, drum beats held twice weekly	SR	8 wks.	Head start classrooms	2 assistant teachers, researcher
20	Webster-Stratton 2008	United States	Incredible Years (IY) Teacher Classroom Management and Child Social and Emotion curriculum (Dinosaur School)	1768 Headstart and elementary school students, average 63.7 mos. age, ethnically diverse	30 classroom lessons per yr. with 300 small group activities, 100 videotaped models of children for socioemotional skills and conflict management strategies.	SR	5 mos.	School	Trained teachers, certified research staff
21	Yoshikawa 2015	Chile	Un Buen Comienzo (A Good Start; UBC)	1876 from 64 prekindergarten and kindergartens in six low-income municipalities	12 modules combining didactic (content-focused strategies) and coaching components for teachers, and provision of a library of roughly 100 books per classroom.	SR, classroom organization	2 yrs.	Kindergarten	Trained coaches

© 2018 Anuja Pandey, MD, et al. *JAMA Pediatrics.*

Abbreviations: SR=Self-regulation;SD=standard deviation;mos.=months;wks.=weeks;yrs.=years;min=minutes;hr.=hour

© 2018 Anuja Pandey, MD, et al. *JAMA Pediatrics.*

eTable 2: Characteristics of Family based interventions including population description, setting and intervention details

Sr. no.	Study ID	Country	Intervention	Population Characteristics	Intervention description	Intervention aim/theory	Duration	Setting	Providers
1	Brody 2005	United States	Strong African American Families (SAAF)	11-yr.olds from working poor households in 9 rural counties	7 Concurrent and family sessions (1 hr. each) held at community facilities, with separate parent and child skill-building curricula and a family curriculum.	self-control, enhance parent-child relationships	7 wks.	Community	Trained team leaders delivered the group intervention
2	Chang 2015	United States	FCU/Family Check Up	731 families with 2 yr. olds from low income group	2 or more sessions with Parent Consultant, 2.5 hr home visit annually at age 2,3 & 4 yrs. to enhance proactive parenting and effortful control	effortful control, proactive parenting	3 yrs.	Community	Parent Consultant
3	De Wit 2007	Canada	BBBS Program (Big Brothers Big Sisters Community Match Program)	71 families (parents and children ages 7–14 yrs.) and 30 adult mentors	One-on-one mentoring program for children from disadvantaged backgrounds, with weekly 2-4 hr. meetings with a caring and responsible adult engaging in leisure & career-oriented activities	social skills (self-control), social anxiety	1 yr.	Community	Trained mentors

© 2018 Anuja Pandey, MD, et al. *JAMA Pediatrics.*

4	Feinberg 2013	United States	Siblings are Special (SIBS)	174 families with siblings in 2nd – 5th grade	Weekly after school sessions (1.5 hrs. each) and three family nights (2 hrs. each) for enhancing youths' socioemotional competencies & parents' ability to manage sibling relationships.	self-control, positive sibling relationships, parental involvement	12 wks.	School	Trained group leaders
5	Fosco 2013	United States	Family Check-Up (FCU)	593 ethnically diverse middle school students	Multilevel program with family resource centre (FRC), in middle schools ,Family Check-Up (FCU): three brief sessions that are grounded in motivational interviewing & Parent consultation components	SR	4 yrs.	School	Parent consultants
**Sr. no.**	**Study ID**	**Country**	**Intervention**	**Population Characteristics**	**Intervention description**	**Intervention aim/theory**	**Duration**	**Setting**	**Providers**
6	Stormshak 2010	United States	Family Check-Up (FCU)	377 adolescents across 3 public middle schools, ethnically diverse	Multilevel program with family resource centre (FRC), in middle schools ,Family Check-Up (FCU): three brief sessions that are grounded in motivational interviewing & Parent consultation components	SR	4 yrs.	School	Parent consultants

© 2018 Anuja Pandey, MD, et al. *JAMA Pediatrics.*

7	Kumpfer 2002	United States	SAFE (Strengthening America's Families and Environment)	1st graders from 12 rural schools	14 session multicomponent family skills training program consisting of three courses: parent skills training, children skills training, and family life skills training, with booster at 6 months & 1 yr.	parenting skills, SR, school bonding,	1 yr.	School	Parent & Child Trainers
8	Mason Alex 2015	United States	Common Sense Parenting	321 8th grade students with more than >75% qualifying for free school lunch	CSP weekly sessions focussed on parenting skills related to discipline, praise, rationales, coping, problem solving, and anger management; short videos & guided skills practice. ,	emotional regulation skills, parenting	6 wks.	Home visits & parent workshops	Trained CSP trainers
9	Sheridan 2010	United States	Getting Ready	220 3-5 yr. old disadvantaged preschool children	The intervention integrates triadic (parent–child–professional) and collaborative (family–school) strategies to promote parent–child and parent–professional partnerships with home visits & parent conferences	socioemotional competencies, school readiness	1 yr.	Preschool	Trained Head Start teachers

Abbreviations: SR=Self-regulation;mos.=months;wks.=weeks;yrs.=year;hr.=hour

© 2018 Anuja Pandey, MD, et al. *JAMA Pediatrics.*

eTable 3: Characteristics of mindfulness & yoga interventions including population description, setting and intervention details

Sr. no.	Study ID	Country	Intervention	Population Characteristics	Intervention description	Intervention aim/theory	Duration	Setting	Providers
1	Butzer 2017	United States	Yoga	7th grade students in a public school (intervention = 117 & control = 94), with a mean age of 12.64, primarily White and Asian students	A 32-session version of the Kripalu Yoga in the Schools (KYIS) curriculum with 1-2 sessions per wk..	SR, substance use risk	6 mos.	School	Trained teachers with advanced training in the KYIS intervention
2	Flook 2015	United States	Kindness Curriculum(KC)	68 preschool children	Two 20–30 min. lessons each wk. of mindfulness-based prosocial skills training	executive functioning, social competence	12wk.	Preschool	experienced mindfulness instructors
3	Fishben 2016	United States	Mindful Yoga	85 (40 control, 45 intervention) participants (mean age = 16.7 yrs.) from grades 9-12 in a credit recovery school, ethnically diverse	The 20 session (50 min each) mindful yoga curriculum utilizes key mindfulness principles that are practiced and emphasized throughout the sessions.	SR, psychological stress, substance use	7 wks.	School	Yoga instructor and Assistant
4	Flook 2010	United States	Mindful awareness practices (MAPs)	64 second and third-grade children aged 7–9 yrs.	The MAPs training includes 30 min sessions twice a wk., uses secular and age appropriate exercises and games to promote awareness of self ,others and environment.	executive functions	8 wks.	School	Facilitators

© 2018 Anuja Pandey, MD, et al. *JAMA Pediatrics.*

Sr. no.	Study ID	Country	Intervention	Population Characteristics	Intervention description	Intervention aim/theory	Duration	Setting	Providers
5	Mendelson 2010	United States	Yoga-inspired mindfulness program	97 4th and 5th graders from 4 urban public schools; 83.5% African American, 4.1% Latino, 4.1 % White, and 7.2% mixed race	Key intervention components included yoga-based physical activity, breathing techniques, and guided mindfulness practices with 45 min sessions, 4 days/wk.	SR	12 wks.	School	Yoga instructors from same ethnic background as subjects
6	Kimberley 2015	Canada	MindUP Program	4th and 5th graders (N = 99) , age 9-11 yrs. from median annual income neighbourhoods	The curriculum includes 12 lessons (40-50 min each) that promote EFs and SR and positive mood	SR, socioemotional wellbeing	12 wks.	School	Trained Teachers
7	Noggle 2012	United States	Kripalu Yoga	51 students from Grades 11 and 12 at a public high school ,average age 17 yrs.,	28 session yoga intervention with 4 key elements of classical yoga: physical exercises and postures, breathing exercises, deep relaxation, and meditation techniques.	SR, psychological well being	10 wks.	Classroom	Trained yoga teachers and yoga assistants

© 2018 Anuja Pandey, MD, et al. *JAMA Pediatrics.*

8	Parker 2014	United States	Master Mind	4th and 5th grade children (intervention, n=71, control, n=40)	The 20 lesson Master Mind curriculum is divided into four sections and each section represents one of the four foundations of mindfulness (Awareness of Feelings, Awareness of Thoughts, and Awareness of Relationships & Awareness of the Body).	SR	1 mo.	Trained teachers	Trained teachers

Abbreviations: SR=Self-regulation;mo.=month;wks.=weeks;yrs.=years;min=minute

eTable 4: Characteristics of exercise based interventions including population description, setting and intervention details

Sr. no.	Study ID	Country	Intervention	Population Characteristics	Intervention description	Intervention aim/theory	Duration	Setting	Providers
1	Cecchini 2007	Spain	Personal & Social Responsibility model	186 students from 3 public schools with a mean age of 13.6 yrs.	Personal and social responsibility based strategies used during soccer sessions in physical education class	self-control, fair play	2 mos.	School	Trained instructor
2	Chen 2014	China	acute aerobic exercise	98 preadolescents in an elementary school in the Miyun dist., Beijing	group jogging at moderate intensity for 30 min	executive function	1 session	School	Trainers

© 2018 Anuja Pandey, MD, et al. *JAMA Pediatrics.*

3	Costigan 2016	Australia	High-intensity interval training (HIIT)	Grade 9 & 10 students (n = 65; mean age = 15.8 , SD= 0.6 yr)	Participants completed HIIT sessions primarily involving gross motor cardiorespiratory exercises (e.g., shuttle runs, jumping jacks, and skipping).	executive function, psychological well-being, psychological distress, and physical self-concept	3 HIIT sessions per wk. for 8 wk (24 sessions in total)	School	Not described
4	Hillman 2014	United States	FIT Kids physical activity program	221 study participants aged 7-9 yrs.	Children intermittently participated in at least 70-mins. of moderate to vigorous PA including 30 to 40 min. at PA stations followed by rest and organizational games (45–55 min.) centered on a skill theme.	executive control	9 mos.	After school at recreational facility	Not described
**Sr. no.**	**Study ID**	**Country**	**Intervention**	**Population Characteristics**	**Intervention description**	**Intervention aim/theory**	**Duration**	**Setting**	**Providers**
5	Kimberley 2004	United States	Leadership Education Through Athletic Development (LEAD) curriculum.	207 children from kindergarten through Grade 5; 73% families with high income	26 session (45 min. each) Martial Arts Program for self-improvement	SR	4 mos.	School	Martial arts instructor
6	Schmidt 2015	Switzerland	Team games (high cognitive engagement, high physical exertion)	181 children ranging from 10 to 12 yrs. of age	2 physical education lessons (45 min. each) per wk. designed team games (floorball and basketball) tailored to challenge executive function	executive function	6 wks.	School	Trained teachers

© 2018 Anuja Pandey, MD, et al. *JAMA Pediatrics.*

Abbreviations: SR=Self-regulation;SD=standard deviation;mos.=months;wks.=weeks;yrs.=years;min=minutes

eTable 5: Characteristics of social and personal skills interventions including population description, setting and intervention details

Sr. no.	Study ID	Country	Intervention	Population Characteristics	Intervention description	Intervention aim/theory	Duration	Setting	Providers
1	Murray 2015	United Kingdom	Attention Training	100 children (Age range: 5.20 -6.52 yrs.)from 5 primary schools in Greater Manchester	A recorded version of Wells' (1990) Attention Training Technique played on 3 separate occasions	self-regulatory capacities including delayed gratification and executive control	4 days	School	Teachers
2	Saltz 1977	United States	Fantasy Play	80 preschoolers from lower-level economic population, ranging from 3 to 4.5 yrs.	Thematic-fantasy play sessions of 15 min/day, 3 days/wk. using fairy tales, socio dramatic fantasy play using day to day experiences, fantasy discussion	Impulse control	1 wk.	Preschool	Trained staff

© 2018 Anuja Pandey, MD, et al. *JAMA Pediatrics.*

3	Toner 1978	United States	Model Behaviour	90 boys with mean age of 52.2 mos.	Model behaviour training with televised peer models who resisted the temptation to play with attractive, but prohibited toys	self-control	1 session	Preschool	Staff
4	Traverso 2015	Italy	Executive function training	75 kindergarten children (Age, Mean = 68.6 mos; SD=3.5) in disadvantaged areas	30 min sessions of small group game activities challenging inhibitory control, working memory, and cognitive flexibility, thrice a wk.	executive function	1 mo.	Kindergarten	Trained psychologist
**Sr. no.**	**Study ID**	**Country**	**Intervention**	**Population Characteristics**	**Intervention description**	**Intervention aim/theory**	**Duration**	**Setting**	**Providers**
5	Trostle 1998	United States	child-centered group play sessions	48 bilingual Puerto Rican 3 to 6 yr.old children	5 behavioural "tools" during each of the child-centered play sessions for 40 min, once each wk.	self-control	10 wks.	Preschool	university faculty member trained in child centred play
6	Volckaert 2015	Belgium	Inhibition training	47 preschoolers (mean age=60 mos.)	Two 45min. sessions per wk. of exercises/gamestapping on components of inhibition functions	inhibition training	8 wks.	Preschool	Neuropsychologist and a psychology trainee.

Abbreviations: SD=standard deviation;mos.=months;wks.=weeks;yrs.=years;min=minutes

© 2018 Anuja Pandey, MD, et al. *JAMA Pediatrics.*

eTable 6: Effect of self-regulation interventions on health and social outcomes : Name of Intervention, Outcomes and results

Study no.	Study ID	Intervention name	Outcomes	Results
1	Barnett 2008	Tools of the Mind	Academic Achievement	Participation in intervention improved children's academic achievement in language, but these effects were smaller and did not reach conventional levels of statistical significance in multi-level models or after adjustments for multiple comparisons.
2	Blair 2014	Tools of the Mind Curriculum	Academic Achievement	Participation in intervention led to improvements in academic achievement (reading, vocabulary, and mathematics) at the end of first year of intervention, which continued into the next year.
3	Bowers 2015	Student Success Skills Program	Academic Achievement	Results from multiple path analyses indicated that participation in intervention was associated with better academic performance (reading). Effect size=0.34 (95% CI,0.06, 0.62).
4	Flook 2015	Kindness Curriculum	Academic achievement	The intervention group showed better academic performance earning higher grades in three subjects, with no difference in grades compared to controls in two subjects.

© 2018 Anuja Pandey, MD, et al. *JAMA Pediatrics.*

Study no.	Study ID	Intervention name	Outcomes	Results
5	Feinberg 2013	Siblings are Special (SIBS)	Academic achievement	There was statistically significant improvement in academic performance of intervention group compared to the control group.
6	Lemberger 2015	Student Success Skills	Academic achievement (Language, Mathematics)	The intervention group showed gains in academic achievement in mathematics and reading on a high-stakes standardized test, compared to controls.
7	O Connor 2014	INSIGHTS	Academic achievement	Children enrolled in the intervention group experienced growth in math and reading achievement that was significantly faster than that of children enrolled in the supplemental reading program.
8	Pears 2014	Kids in Transition to School (KITS)	Academic Achievement	Children who received the intervention demonstrated significantly greater improvements in academic achievement in areas of letter naming, initial sound fluency, and understanding of concepts about print than their peers who did not participate in the intervention.
9	Raver 2011	Chicago School Readiness Project	Academic Achievement	Participation in intervention was associated with significant benefits in areas of vocabulary, letter-naming, and math skills.

© 2018 Anuja Pandey, MD, et al. *JAMA Pediatrics.*

Study no.	Study ID	Intervention name	Outcomes	Results
10	Riggs 2006	PATHS Curriculum	Academic achievement	The intervention group showed better academic achievement in area of verbal fluency compared to control group.
11	Kimberley 2004	LEAD Martial Arts	Academic Achievement	Intervention group showed improved performance on performance on a mental math test.
12	Tominey 2011	Red Light, Purple Light	Academic achievement	Children in the intervention group demonstrated significant academic achievement (letter-word identification) gains compared to children in the control group.
13	Yoshikawa 2015	UBC	Academic achievement	There was no significant impact of intervention on academic achievement (language and literacy skills).
14	Butzer 2017	Yoga	Substance use	There were significant beneficial effects, with a reduction in substance use (willingness to smoke) in the intervention group compared to controls.
15	Brody 2005	Strong African American Families Program(SAAF)	Substance use	There was significant reduction in substance use (alcohol) in the intervention group compared to controls at 65 month assessment. The alcohol intake for intervention group was almost half of that of control group.

© 2018 Anuja Pandey, MD, et al. *JAMA Pediatrics.*

Study no.	Study ID	Intervention name	Outcomes	Results
16	Fosco 2013	Family Check Up (FCU)	Substance Use	Participation in Family Check Up intervention reduced the risk for alcohol, tobacco, and marijuana use over follow up period of three yrs.
17	Mason Alex 2016	Common Sense Parenting	Substance Use	The intervention had statistically significant indirect effects on reduced substance use (past-year use of alcohol or marijuana and past-month use of cigarettes) at the 1-yr. follow-up.
18	Parker 2014	Master Mind	Substance Use	No significant differences across groups were found for intentions to use alcohol or tobacco.
19	Brody 2005	Strong African American Families Program(SAAF)	Conduct Problems	Intervention youth were less involved than control-group youth in conduct problems across time.
20	Mason Alex 2016	Common Sense Parenting	Conduct Problems	The intervention had statistically significant indirect effects on conduct problems at the 2-yr. follow-up
21	Webster-Stratton 2008	Incredible Years (Dinosaur School)	Conduct Problems	The study showed fewer conduct problems in intervention group compared to the control group.
22	O Connor 2014	INSIGHTS	Behavior Problems	Children participating in the intervention showed decreases in behavior problems over

© 2018 Anuja Pandey, MD, et al. *JAMA Pediatrics.*

Study no.	Study ID	Intervention name	Outcomes	Results
				time, while children in control group demonstrated increases.
23	Fosco 2013	Family Check Up (FCU)	Behavior Problems	Participation in Family Check Up intervention reduced the risk for growth in antisocial behaviour, involvement with deviant peers over follow up period of three yrs.
24	Clarke 2014	Zippy's Friends	Social skills	There was significant positive effect on self-awareness, motivation and social skills in intervention group compared to controls.
25	De Wit 2007	BBBS Program (Big Brothers Big Sisters Community Match Program)	Social Skills	There was significant benefit in social skills in intervention group compared to controls. (symptoms of emotional problems, symptoms of social anxiety).
26	Stormshak 2010	Family Check Up	Mental Health(Depression)	There was statistically significant decrease in intervention group compared to controls.
27	Kimberley 2015	Mind UP	Mental Health	Subjects in intervention group showed greater decreases in self-reported symptoms of depression and peer-rated aggression.
28	Mason Alex 2016	Common Sense Parenting	School suspensions	Intervention had statistically significant indirect effects on reduced school suspensions at one and two year follow-up

© 2018 Anuja Pandey, MD, et al. *JAMA Pediatrics.*

Study no.	Study ID	Intervention name	Outcomes	Results
29	Costigan 2016	Aerobic Exercise Program	Psychological Wellbeing	While the results were not significant, there was small change in psychological wellbeing in the intervention group compared to controls.

Abbreviations: CI=Confidence interval; yr.=year

© 2018 Anuja Pandey, MD, et al. *JAMA Pediatrics.*

Efigure 1:Funnel Plot for pooled analysis of effect sizes of self-regulation task performance scores

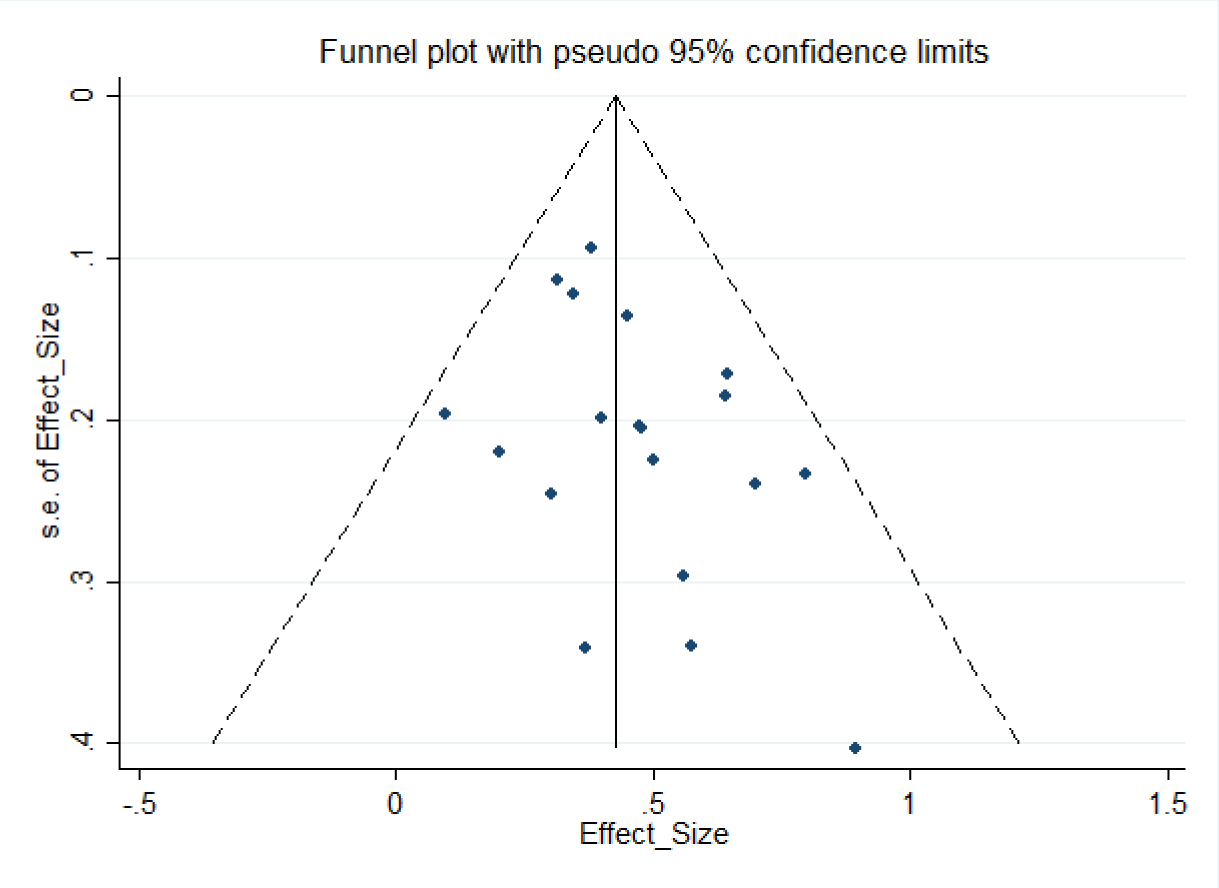

© 2018 Anuja Pandey, MD, et al. *JAMA Pediatrics.*
